# Beneficial Antioxidant Effects of Coenzyme Q10 in In Vitro and In Vivo Models of CDKL5 Deficiency Disorder

**DOI:** 10.3390/ijms26052204

**Published:** 2025-02-28

**Authors:** Manuela Loi, Francesca Valenti, Giorgio Medici, Nicola Mottolese, Giulia Candini, Angelica Marina Bove, Federica Trebbi, Luca Pincigher, Romana Fato, Christian Bergamini, Stefania Trazzi, Elisabetta Ciani

**Affiliations:** 1 Department of Biomedical and Neuromotor Sciences, University of Bologna, Piazza di Porta San Donato 2, 40126 Bologna, Italy; manuela.loi3@unibo.it (M.L.); giorgio.medici2@unibo.it (G.M.); nicola.mottolese2@unibo.it (N.M.); giulia.candini4@unibo.it (G.C.); angelicamarina.bove2@unibo.it (A.M.B.); federica.trebbi3@unibo.it (F.T.); elisabetta.ciani@unibo.it (E.C.); 2 Department of Pharmacy and Biotechnology, University of Bologna, Via Irnerio 42, 40126 Bologna, Italy; francesca.valenti8@unibo.it (F.V.); luca.pincigher2@unibo.it (L.P.); romana.fato@unibo.it (R.F.); christian.bergamini2@unibo.it (C.B.)

**Keywords:** *CDKL5* deficiency disorder, oxidative stress, coenzyme Q10, mouse model

## Abstract

*CDKL5* deficiency disorder (CDD), a developmental encephalopathy caused by mutations in the cyclin-dependent kinase-like 5 (*CDKL5*) gene, is characterized by a complex and severe clinical picture, including early-onset epilepsy and cognitive, motor, visual, and gastrointestinal disturbances. This disease still lacks a medical treatment to mitigate, or reverse, its course and improve the patient’s quality of life. Although CDD is primarily a genetic brain disorder, some evidence indicates systemic abnormalities, such as the presence of a redox imbalance in the plasma and skin fibroblasts from CDD patients and in the cardiac myocytes of a mouse model of CDD. In order to shed light on the role of oxidative stress in the CDD pathophysiology, in this study, we aimed to investigate the therapeutic potential of Coenzyme Q10 (CoQ10), which is known to be a powerful antioxidant, using in vitro and in vivo models of CDD. We found that CoQ10 supplementation not only reduces levels of reactive oxygen species (ROS) and normalizes glutathione balance but also restores the levels of markers of DNA damage (γ-H2AX) and senescence (lamin B1), restoring cellular proliferation and improving cellular survival in a human neuronal model of CDD. Importantly, oral supplementation with CoQ10 exerts a protective role toward lipid peroxidation and DNA damage in the heart of a murine model of CDD, the *Cdkl5* (+/−) female mouse. Our results highlight the therapeutic potential of the antioxidant supplement CoQ10 in counteracting the detrimental oxidative stress induced by CDKL5 deficiency.

## 1. Introduction

*CDKL5* (cyclin-dependent kinase-like 5) deficiency disorder (CDD) is a rare and severe X-linked developmental encephalopathy, caused by mutations in the *CDKL5* gene [1,2,3], with an estimated incidence of 1:40,000–60,000 live births [4,5] and a female-to-male ratio of 4:1. The clinical features of CDD include severe neurological symptoms, such as early-onset seizures, intellectual disability, cortical visual impairment, poor sleep, and gross motor impairment [4,6]. The improvement of the clinical overview of CDD in the past few years has defined a more detailed phenotypic spectrum; this includes very common alterations in peripheral organ and tissue function, such as gastrointestinal problems, irregular breathing, hypotonia, and scoliosis [7].

*CDKL5* encodes a serine-threonine kinase that is highly expressed in neurons [8], particularly in axons, dendrites, and spines [9,10]. Studies in mouse models of CDD have shown that loss of CDKL5 in the central nervous system (CNS) results in behavioral deficits across motor, sensory, cognitive, and social-emotional domains that are reminiscent of human symptomatology [11,12,13,14,15]. Various studies of *Cdkl5* mouse models and *CDKL5*-deficient neuronal cells converge on the role of CDKL5 in neuronal survival, dendritic arborization, axonal outgrowth, and synaptic function [6,16,17,18]. The role of CDKL5 in biological processes that take place in non-neuronal tissues has recently emerged. Indeed, a recent study described cardiac functional and structural abnormalities in heterozygous *Cdkl5* +/− female mice [19]. *Cdkl5* +/− mice exhibited QTc prolongation and increased heart rate accompanied by typical signs of heart aging, including increased fibrosis, mitochondrial dysfunctions, and increased ROS production. A similar accelerated aging due to neuronal senescence, associated with increased amounts of damaged DNA [20] and mitochondrial functional abnormalities [21,22], was observed in the Cdkl5-null brain. These findings are in line with evidence that redox imbalance occurs in plasma from CDD patients [23,24,25,26].

Oxidative stress is a combination of biochemical events resulting in damage to biological molecules because of an imbalance between cellular antioxidant defenses and ROS production. Converging data from animal models and human research indicate that oxidative stress likely represents a shared feature present in many brain disorders and, more specifically, in neurodevelopmental disorders (NDDs) [27,28]. For instance, Rett syndrome (RTT) is associated with redox alterations, an oxidant burden, and oxidative tissue damage [29,30,31], and treatments with antioxidants and free-radical scavengers ameliorate certain aspects of its complex and severe clinical presentation [32,33].

Among the antioxidants, Coenzyme Q10 (CoQ10), a vitamin-like substance formed by endogenous synthesis [34], has a key role as an electron carrier in the mitochondrial respiratory chain activity. Moreover, it is a crucial, lipid-soluble, radical-trapping antioxidant in the cellular membrane, and it protects the mitochondrial and extra-mitochondrial membrane from free radicals [35,36,37]. CoQ10 has been extensively used in the medical field; its therapeutic applications range from neurodegenerative diseases such as multiple system atrophy (MSA) to conditions such as Barth syndrome, heart failure, fibromyalgia, or insulin resistance [38]. Furthermore, oral treatment with CoQ10 significantly attenuates the oxidative stress-induced damage in neurodevelopmental disorders such as RTT [32,33], Down syndrome [39], and autism spectrum disorders [40]. Despite the potential benefits of CoQ10 supplementation, the clinical use of this potent antioxidant is hindered by its low absorption and tissue distribution [41]. In order to face these problems, many formulations have been developed to ameliorate CoQ10 bioavailability. CoQ10 Phytosome formulation (Ubiqsome or UBQ) [42] led to a significant increase in quinone level in both cell and mitochondrial lysate [43]. Furthermore, it has been reported that oral administration of UBQ improved the total CoQ10 amount in skeletal muscle tissue [44], indicating that Phytosome carriers provide a great advantage in in vivo CoQ10 delivery.

Based on the evidence that increased oxidative stress may have a causative effect in the CDD phenotype, this study focused on investigating the effect of UBQ supplementation to rescue CDKL5 deficiency-dependent phenotypes in an in vitro and in vivo model of CDD. We examined the efficacy of treatment with UBQ to rescue ROS production, glutathione balance, and cellular proliferation/survival in a human neuronal model of CDD and in promoting antioxidant defense in the heart of a murine model of CDD, the *Cdkl5* knockout (KO) mouse.

## 2. Results

### 2.1. Treatment with UBQ Has a Protective Effect Against Increased Oxidative Stress in a Human Cellular Model of CDKL5 Deficiency

Since oxidative stress is a characteristic trait of the CDD phenotype, we evaluated the effect of UBQ supplementation on oxidative stress in a human neuronal cell model of CDKL5 deficiency, the CDKL5 knockout (KO) SH-SY5Y neuroblastoma cell line (SH*-CDKL5-*KO [18]). While we observed no differences in CoQ10 endogenous synthesis between SH-SY5Y and CDKL5 KO cells (two-way ANOVA genotype effect: F (1, 11) = 0.3782, *p* = 0.55; Table S1), treatment with UBQ increased CoQ10 levels in both wild-type and SH-CDKL5 KO cells (two-way ANOVA treatment effect: F (1, 11) = 15.99, *p* = 0.002; Table S1), demonstrating efficient CoQ10 cell intake.

**Figure 1:**
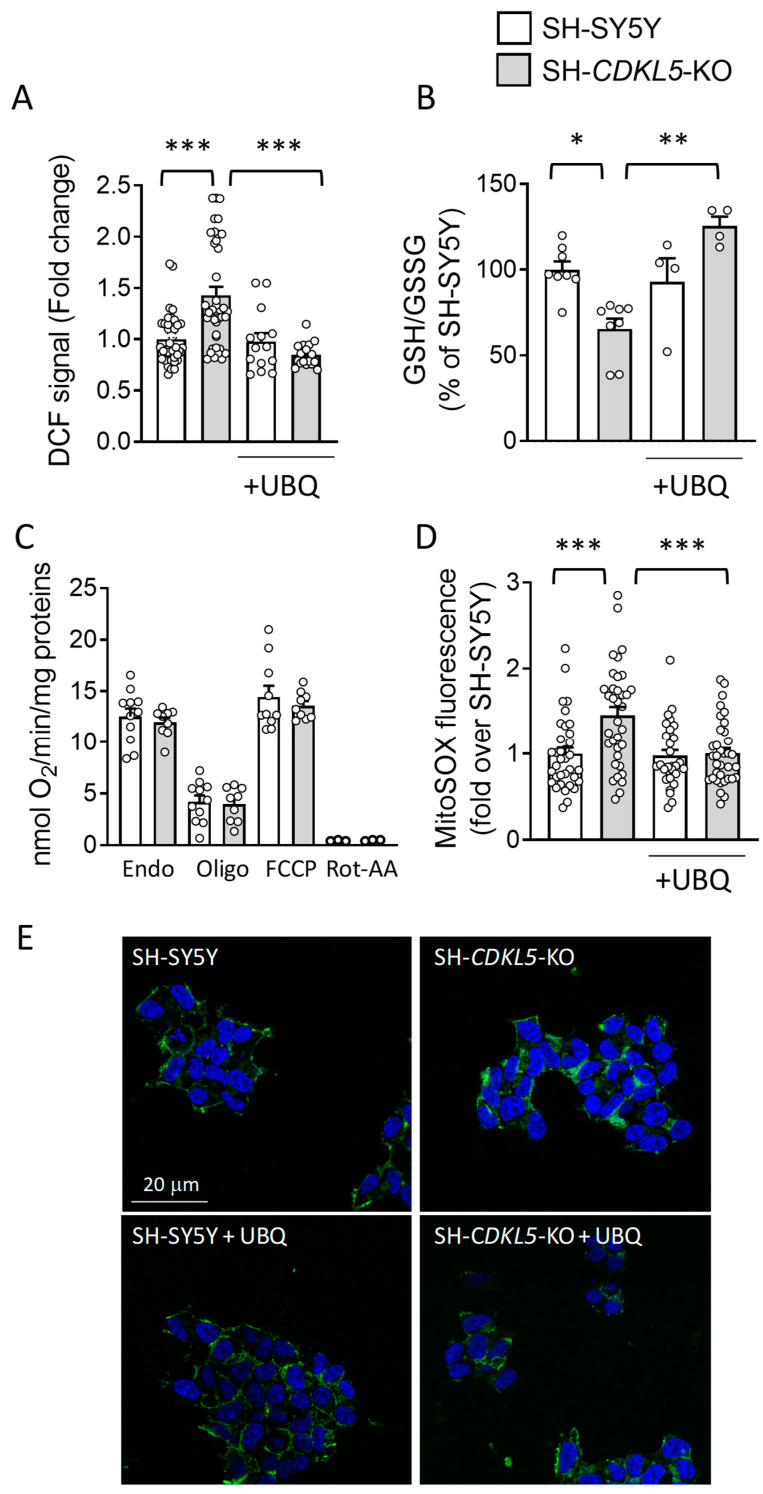
Effect of treatment with UBQ on ROS production/oxidative stress in SH*-CDKL5-*KO cells. (**A**) Oxidative stress determination in SH-SY5Y and SH*-CDKL5-*KO cells. The cells were incubated with 100 nM CoQ10 Phytosome (UBQ) or vehicle for 24 h. Endogenous oxidative stress (**A**) was measured in intact SH-SY5Y and SH*-CDKL5-*KO cells using the fluorescent probe 2′,7′-dichlorodihydrofluorescein diacetate (DCF). The data were normalized to protein content and expressed as fluorescence signal fold change relative to vehicle-treated SH-SY5Y control cells. (**B**) Evaluation of reduced/oxidized glutathione ratio (GSH/GSSG) in SH-SY5Y and SH*-CDKL5-*KO treated as in (**A**). The data were normalized to protein content and presented as a percentage of vehicle-treated SH-SY5Y control cells. (**C**) Mitochondrial oxygen consumption rate in intact SH-SY5Y and SH*-CDKL5-*KO neuroblastoma cells (endogenous respiration “Endo”), in the presence of 1 µM oligomycin A (Oligo), 0.25–1 µM carbonyl cyanide 4-(trifluoromethoxy) phenylhydrazone (FCCP), and 100 nM Rotenone and 100 nM AntimycinA (Rot-AA). The data are expressed as nanomoles of oxygen per minute and normalized to protein content. (**D**) Oxidative stress determination in mitochondria of SH-SY5Y and SH*-CDKL5-*KO cells treated as in (**A**), using MitoSOX Green. Approximately 35–40 cells were analyzed for each experimental condition. (**E**) Representative images of cells stained with MitoSOX Green and DAPI (blue). The values in (**A**–**D**) represent a mean ± SEM of at least 3 independent experiments. * *p* < 0.05; ** *p* < 0.01; *** *p* < 0.001 (Tukey test after two-way ANOVA).

Using the fluorogenic probe 2′,7′-dichlorodihydrofluorescein diacetate (DCF-DA), we found a significantly higher ROS production in SH*-CDKL5-*KO cells in comparison with parental SH-SY5Y cells (Figure 1A).

Treatment with UBQ (100 nM) reduced the total amount of cellular ROS in SH*-CDKL5-*KO cells at the level of the parental cells (Figure 1A). The oxidative status in parental SH-SY5Y cells was not affected by UBQ treatment (SH, Figure 1A). SH*-CDKL5-*KO cells showed a decreased reduced/oxidized glutathione ratio (GSH/GSSG) compared to parental cells, confirming a higher oxidative stress status. Treatment with UBQ significantly increased the GSH/GSSG ratio in KO cells, at levels even higher than those of the parental cells, suggesting a protective effect, whereas no effect was observed in parental cells (Figure 1B).

To investigate whether differences in ROS levels are related to mitochondrial respiratory chain impairment, oxygen consumption rates (OCRs) were measured in intact SH*-CDKL5-*KO and parental cells. No differences in OCR were detected under basal conditions, after the addition of oligomycin A (to block the ATPase), of the uncoupler carbonyl cyanide 4-(trifluoromethoxy) phenylhydrazone (FCCP, to achieve maximal oxygen consumption rate) or of Rotenone plus AntimycinA (Rot-AA, to block mitochondrial respiration) in SH*-CDKL5-*KO cells in comparison with parental cells (Figure 1C). The contribution of proton leak to the oxygen consumption was not different between SH*-CDKL5-*KO and parental cells (8.02 ± 0.52, 8.34 ± 0.42, respectively). Taken together, these data suggest that mitochondrial oxygen consumption is not altered by the absence of CDKL5. Similarly, in SH*-CDKL5-*KO and parental cells differentiated by treatment with retinoic acid (RA; Figure S1A), no differences in OCR were observed (Figure S1B).

Interestingly, mitochondria from SH*-CDKL5-*KO cells showed higher ROS production, as evaluated by the fluorogenic probe MitoSox Green, which specifically detects mitochondrial superoxide [45], compared to parental cells (Figure 1D,E), consistent with a state of oxidative stress. Treatment with UBQ reduced the total amount of mitochondrial ROS in SH*-CDKL5-*KO cells at the level of the parental cells (Figure 1D,E).

### 2.2. Treatment with UBQ Restores Lamin B1 Levels and Nuclear Shape in a Human Cellular Model of CDKL5 Deficiency

**Figure 2:**
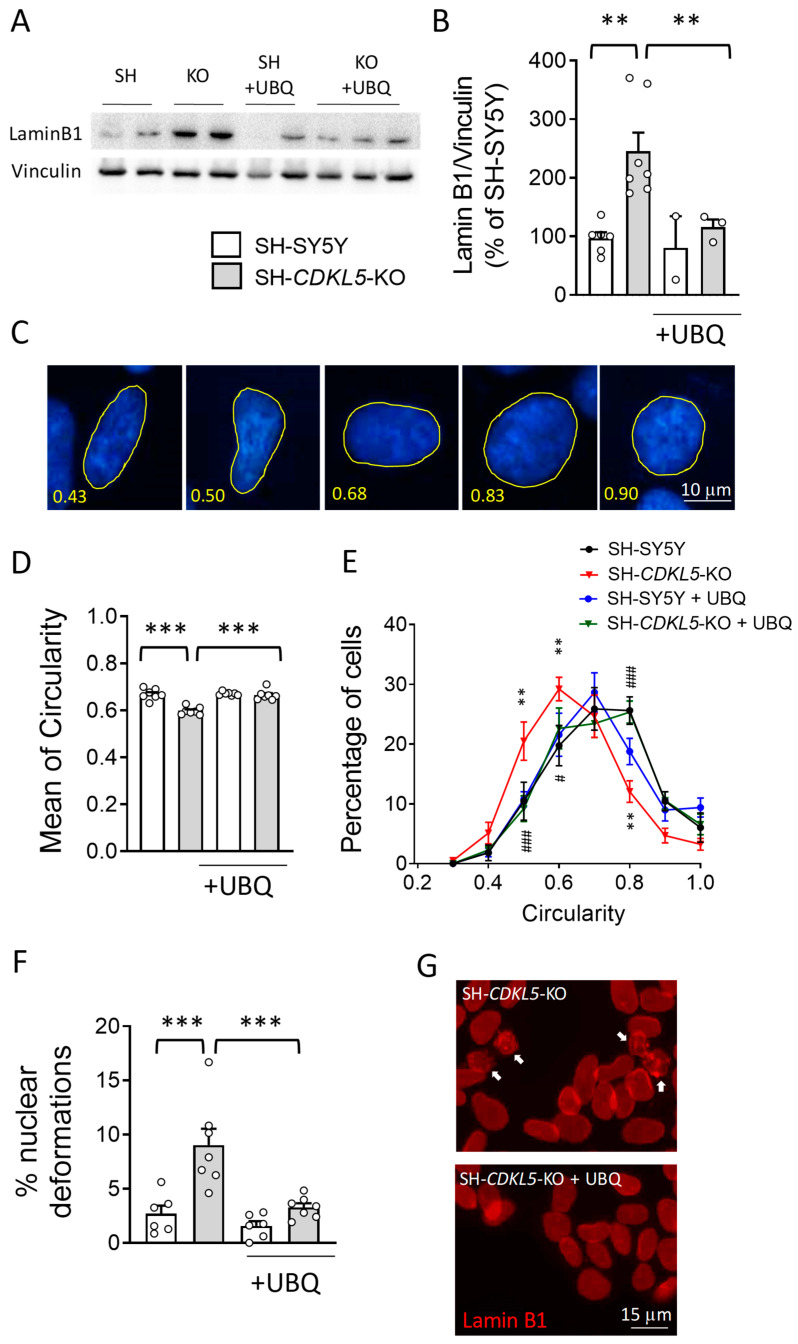
Effect of treatment with UBQ on lamin B1 levels and nuclear shape in SH*-CDKL5-*KO cells. (**A,B**) Expression of lamin B1 in protein extracts from SH-SY5Y and SH*-CDKL5-*KO cells treated with 100 nM CoQ10 Phytosome (UBQ) or vehicle for 24 h. The immunoblots in (**A**) are examples from two/three biological replicates of each experimental condition. The histogram in (**B**) shows quantification of lamin B1 protein levels normalized to vinculin levels. The data are expressed as a percentage of vehicle-treated parental cells. (**C**) Representative fluorescence images of nuclear shape of DAPI-stained neuroblastoma cell nuclei corresponding to different circularity index. (**D**,**E**) Morphometric analysis of nuclei from cells treated as in (**A**). The histogram in (**D**) shows quantification of the mean circularity index. Distribution analysis of nuclear circularity in (**E**). Approximately 200 nuclei were analyzed for each experimental condition. The data are shown as a percentage of nuclei displaying a specific circularity index for each experimental group. (**F**,**G**) Nuclear deformation analysis of lamin B1-stained nuclei from cells treated as in (**A**). The histogram in (**F**) shows the percentage of nuclear deformations for each experimental group. Approximately 200 nuclei were analyzed for each experimental condition. Representative fluorescence images of the nuclear lamin B1 shape appearance of SH*-CDKL5-*KO and SH*-CDKL5-*KO UBQ-treated cells in (**G**). The white arrows indicate nuclei showing morphological aberrations. Approximately 120–150 nuclei were analyzed for each experimental condition. The results in (**B**,**D**–**F**) are presented as means ± SEM. ** *p* < 0.01; *** *p* < 0.001 as compared to the SH-SY5Y condition, # *p* < 0.05; ### *p* < 0.001 as compared to the SH*-CDKL5-*KO condition (Fisher’s LSD test after two-way ANOVA).

Emerging evidence suggests that oxidative stress induces accumulation of the key nuclear architecture component lamin B1 [46]. Western blot analysis showed increased lamin B1 levels in SH*-CDKL5-*KO cells in comparison with parental SH-SY5Y cells (Figure 2A,B). Interestingly, treatment with UBQ restored lamin B1 levels in SH*-CDKL5-*KO cells to the parental cell conditions (Figure 2A,B), suggesting that lamin B1 overexpression in SH*-CDKL5-*KO cells correlates with high ROS production.

Since lamins are major constituents of the inner nuclear membrane and determine its shape and integrity [47,48], nuclear size was determined using DAPI staining. We found that in the absence of CDKL5, while the mean nuclear size was not different (SH-SY5Y: 117.69 ± 6.87 µm^2^, SH*-CDKL5-*KO: 116.22 ± 5.47 µm^2^; *p* = 0.665), nuclear circularity (Figure 2D) was significantly lower in SH*-CDKL5-*KO cells in comparison with parental cells (Figure 2D,E and Figure S2A). The mean circularity measured in SH*-CDKL5-*KO nuclei was significantly lower than the value of 0.65, in contrast with parental cells (Figure 2D). It is important to note that the alteration of the nuclear shape was reverted by treatment with UBQ (Figure 2D,E and Figure S2A). To confirm that the beneficial effect of CoQ10 on nuclear shape in CDKL5-deficient cells was due to its antioxidant action, we tested the effect of a flavonoid, luteolin, a well-known antioxidant with ROS scavenging activities [49,50,51]. We found that luteolin treatment restored alteration of the nuclear circularity in SH-CDKL5 KO cells (Figure S2B,C).

We next asked whether alterations of the nuclear circularity could be associated with nuclear lamina abnormalities. Nuclear deformations such as invaginations, evaginations, and aberrations (Figure 2F,G) were quantified in lamin B1-positive SH*-CDKL5-*KO and parental cells. We observed a reduced proportion of regular nuclei in SH*-CDKL5-*KO cells in comparison with parental cells (Figure 2F). This was mainly due to the increased incidence of nuclear deformations in these cells. Again, the nuclear alteration was reverted by treatment with UBQ (Figure 2F,G), whereas no effect was observed in parental cells (Figure 2F).

### 2.3. Treatment with UBQ Restores Biological Markers Associated with DNA Damage in a Human Cellular Model of CDKL5 Deficiency

**Figure 3:**
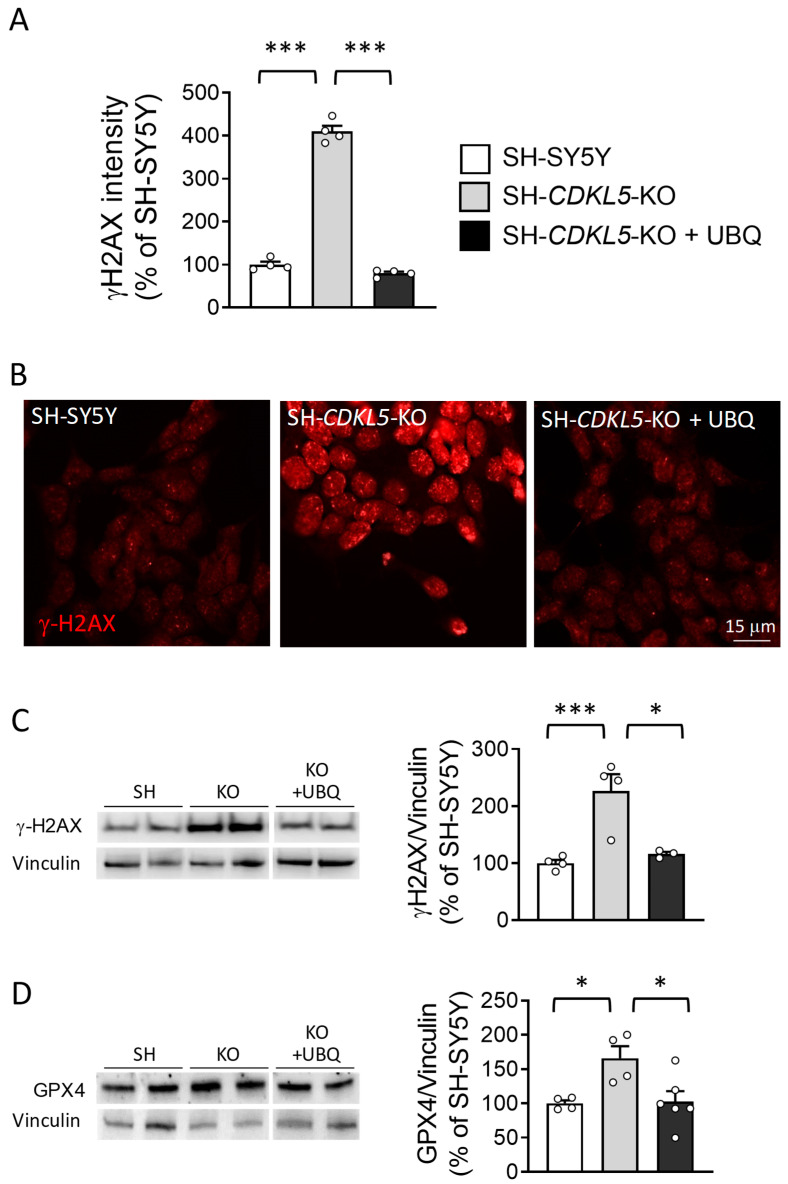
Effect of treatment with UBQ on DNA damage marker γH2AX levels in SH*-CDKL5-*KO cells. (**A**,**B**) Quantification of the intensity of γH2AX nuclear levels in SH-SY5Y and SH*-CDKL5-*KO cells, and in SH*-CDKL5-*KO cells treated with 100 nM CoQ10 Phytosome (UBQ) for 24 h. The histogram in (**A**) shows the mean intensity of nuclear γH2AX staining for each experimental group. Approximately 100 nuclei were analyzed for each experimental condition. Representative fluorescence images of γH2AX-stained nuclei for each experimental group in (**B**). The data are expressed as a percentage of vehicle-treated parental cells. (**C**,**D**) Western blot analysis of γH2AX (**C**) and glutathione peroxidase 4 (GPX4, (**D**)) levels in protein extracts from parental cells (SH-SY5Y), SH*-CDKL5-*KO cells, and SH*-CDKL5-*KO cells treated as in (**A**). The immunoblots are examples from two biological replicates of each experimental condition. The histograms on the right show γH2AX, and GPX4 protein levels normalized to vinculin protein levels. The data are expressed as a percentage of vehicle-treated parental cells. The results are presented as means ± SEM. * *p* < 0.05; *** *p* < 0.001 (Tukey test after one-way ANOVA).

The alteration of the nuclear shape is generally associated with DNA damage and senescence. To explore the possibility that increased DNA damage in SH*-CDKL5-*KO cells [18] might underlie the increased amounts of cellular ROS, we analyzed the levels of DNA damage marker phosphorylated histone H2AX (γH2AX). As previously reported [18], γH2AX levels were significantly higher in SH*-CDKL5-*KO cells compared to parental cells (Figure 3A–C); treatment with UBQ reduced γH2AX levels in SH*-CDKL5-*KO cells at the level of parental cells (Figure 3A–C).

Confirming the increased ROS levels in SH*-CDKL5-*KO cells, we found that glutathione peroxidase 4 (GPX4) levels, which are indicative of increased oxidative stress, were higher in SH*-CDKL5-*KO in comparison with parental cells (Figure 3D). UBQ treatment reduced GPX4 levels in SH*-CDKL5-*KO cells at the level of parental cells (Figure 3D). Differently, the levels of superoxide dismutase 1 (SOD1) and Poly(ADP-ribose) polymerase 1 (PARP1) were no different between parental and SH*-CDKL5-*KO cells, and not affected by UBQ treatment (Table S2).

### 2.4. Treatment with UBQ Restores Neuronal Proliferation and Survival of a Human Cellular Model of CDKL5 Deficiency

**Figure 4:**
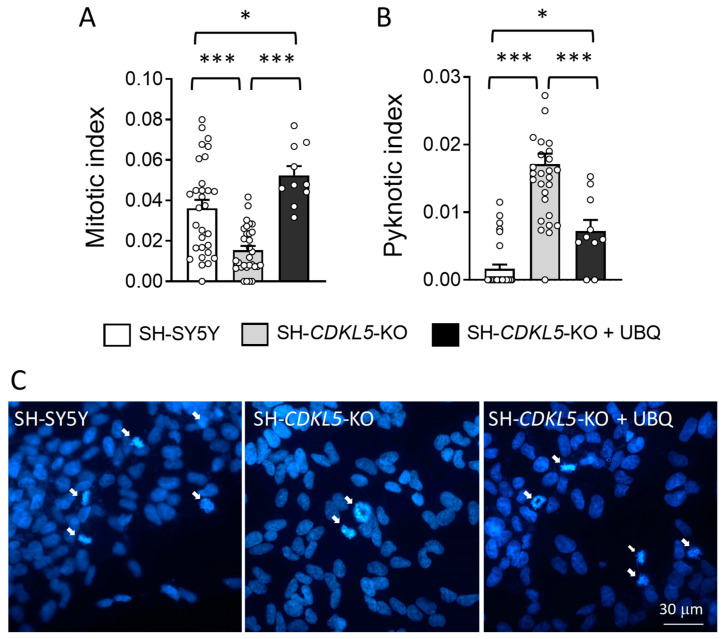
Effect of treatment with UBQ on neuronal proliferation and survival in SH*-CDKL5-*KO cells. (**A**,**B**) Evaluation of the number of mitotic (**A**) and apoptotic (**B**) cells in proliferating vehicle-treated SH-SY5Y and SH*-CDKL5-*KO cells, and in SH*-CDKL5-*KO cells treated with 100 nM UBQ for 24 h. Approximately 120 cells were analyzed for each experimental condition. The data are expressed as mitotic or pyknotic index, i.e., number of mitotic or apoptotic cells over total cell number. (**C**) Representative images showing mitotic nuclei (white arrows) in each experimental condition. The values represent a mean ± SEM of 3 independent experiments. * *p* < 0.05; *** *p* < 0.001 (Tukey test after one-way ANOVA).

When DNA damage is too severe, cells can undergo permanent cell-cycle arrest or cell death [52]. To investigate cell proliferation and viability in SH*-CDKL5-*KO clones, we evaluated the percentage of mitotic and pyknotic nuclei visualized with DAPI staining. As previously reported [18], SH*-CDKL5-*KO cells showed a reduced number of mitotic cells (Figure 4A,C) and an increased number of pyknotic nuclei (Figure 4B) compared to parental cells. Interestingly, we found that treatment with UBQ restored cell proliferation (339 ± 6.1% mitotic cells vs. SH*-CDKL5-*KO cells; Figure 4A,C) and improved survival (42 ± 5.2% pyknotic cells vs. SH*-CDKL5-*KO cells; Figure 4B) in SH*-CDKL5-*KO cells. Similarly, a pro-proliferation and pro-survival effect was observed following luteolin treatment in SH*-CDKL5-*KO cells (Figure S3A–C), confirming the beneficial antioxidant effect of CoQ10.

### 2.5. Treatment with UBQ Decreases ROS Production in the Hearts of Cdkl5 +/− Mice

**Figure 5:**
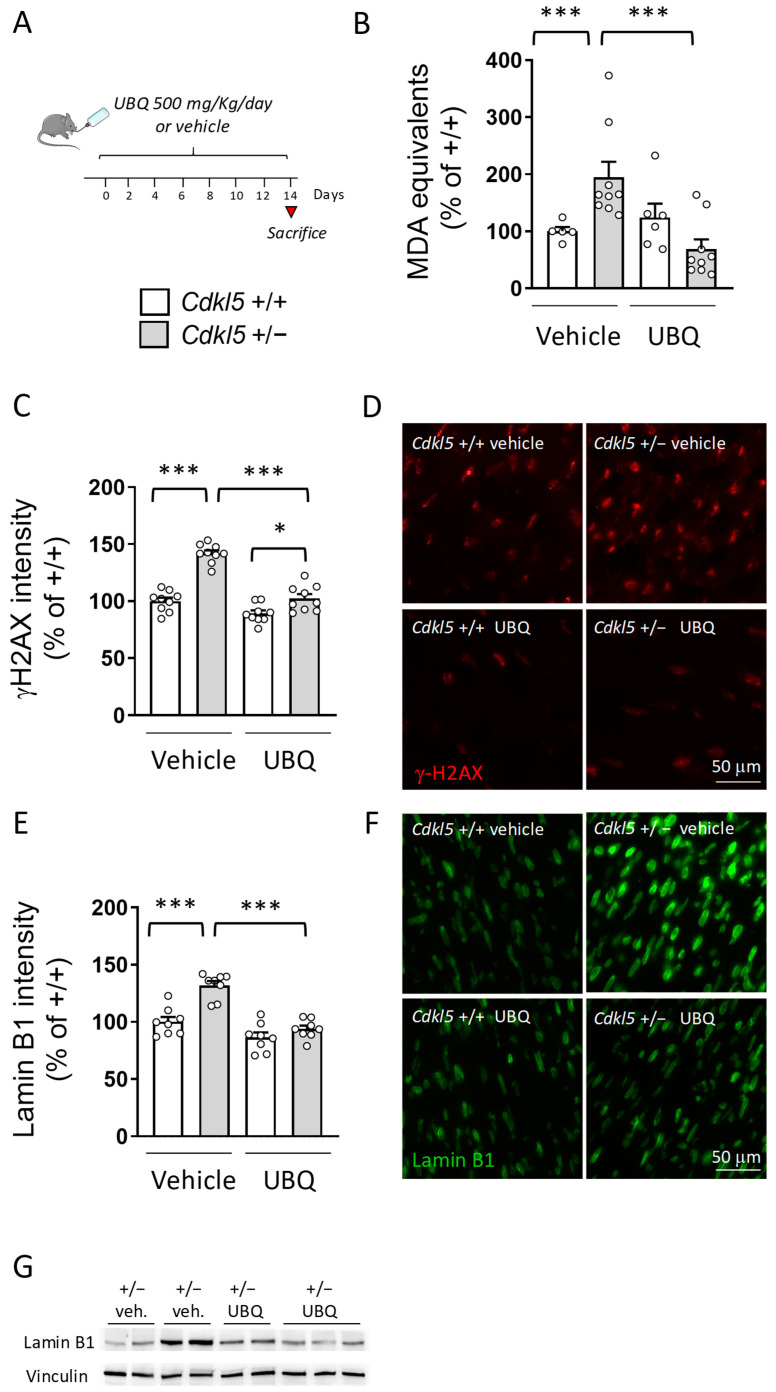
Effect of in vivo treatment with UBQ on ROS production in the hearts of *Cdkl5* +/− mice. (**A**) Schedule of treatment. Six-month-old *Cdkl5* +/+ and *Cdkl5* +/− female mice were provided with drinking water containing CoQ10 Phytosome (UBQ) or Phytosome (vehicle) for two weeks. The daily intake of UBQ for each mouse was equivalent to ~500 mg of UBQ (corresponding to 100 mg of CoQ10) per kilogram of body weight. The mice were sacrificed at the end of treatment. (**B**) Measurement of the levels of lipid peroxidation biomarker malondialdehyde (MDA) in heart homogenates from *Cdkl5* +/+ (n = 5) and *Cdkl5* +/− (n = 9) mice treated with vehicle, and in *Cdkl5* +/+ (n = 6) and *Cdkl5* +/− mice (n = 9) treated with UBQ 500 mg/kg/day for 14 days. The data are expressed as MDA equivalents in percentage of vehicle-treated *Cdkl5* +/+ mice. (**C**,**D**) Quantification of the intensity of γH2AX nuclear levels in cardiomyocytes from *Cdkl5* +/+ (n = 3) and *Cdkl5* +/− mice (n = 3) treated with vehicle, and in *Cdkl5* +/+ (n = 3) and *Cdkl5* +/− mice (n = 3) treated with UBQ 500 mg/kg/day for 14 days. The histogram in (**C**) shows the mean intensity of nuclear γH2AX staining for each experimental group. The data are expressed as γH2AX nuclear intensity in percentage of vehicle-treated *Cdkl5* +/+ mice. Representative fluorescence images of γH2AX-stained nuclei for each experimental group in (**D**). (**E**,**F**) Quantification of lamin B1 intensity in the nuclear envelope of cardiomyocytes from mice as in (**D**). The histogram in (**E**) shows the mean intensity of lamin B1 staining for each experimental group. The data are expressed as lamin B1 intensity in percentage of vehicle-treated *Cdkl5* +/+ mice. Representative fluorescence images of lamin B1-stained nuclei for each experimental group in (**F**). (**G**) Expression of lamin B1 in protein extracts from cardiac tissue of *Cdkl5* +/+ (n = 5) and *Cdkl5* +/− mice (n = 5) treated with vehicle (veh), and in *Cdkl5* +/+ (n = 5) and *Cdkl5* +/− mice (n = 5) treated with UBQ 500 mg/kg/day for 14 days. The immunoblots are examples from two biological replicates of each experimental condition. The results in (**B**,**C**,**E**) are presented as means ± SEM. * *p* < 0.05; *** *p* < 0.001 (Fisher’s LSD test after two-way ANOVA).

Previously, we showed that heterozygous *Cdkl5* KO (*Cdkl5* +/−) female mice are characterized by typical signs of heart aging, including mitochondrial dysfunctions and increased ROS production [19]. To investigate the effect of CoQ10 supplementation on ROS production and oxidative cell stress in the *Cdkl5* +/− heart, UBQ (500 mg/kg) was administered by means of addition to drinking water in adult *Cdkl5* +/− and *Cdkl5* +/+ female mice (6–8-month-old) for two weeks (Figure 5A). A group of vehicle-treated *Cdkl5* +/− and *Cdkl5* +/+ female mice were used as treatment controls. The estimation of daily consumption of water for the vehicle- and UBQ-treated groups showed no treatment or genotype differences (4.5 ± 0.5 mL/day), suggesting a similar UBQ intake between *Cdkl5* +/+ and *Cdkl5* +/− mice. By comparing the *Cdkl5* mRNA levels in heterozygous *Cdkl5 KO (*+/−) hearts to those of wild types, we found, as expected, that the levels in *Cdkl5* +/− mice were lower (about 50%) than those of wild-type mice (Figure S4A,B). Cdkl5 expression levels in *Cdkl5* KO mice were not affected by UBQ treatment (Figure S4A,B).

Measurement of plasma concentrations of CoQ homologues (CoQ9 and CoQ10) showed that CoQ9 and CoQ10 constituted 80% and 20%, respectively, of the CoQ present in the plasma of both wild-type (*Cdkl5* +/+) and *Cdkl5* +/− mice (Table 1), indicating no genotype-dependent differences in CoQ endogenous synthesis. Notably, while intake of UBQ increased the amounts of both CoQ9 and CoQ10 (267% and 183%, respectively) in the plasma of wild-type mice (*Cdkl5* +/+, Table 1), the increase was only marginal and not significant in *Cdkl5* +/− mice (CoQ9: 120%, CoQ10: 112%; Table 1). Otherwise, non-significant increases in CoQ9 and CoQ10 levels were detected in the heart tissues of both UBQ-treated wild-type (*Cdkl5* +/+) and *Cdkl5* +/− mice (Table 2).

As previously reported [19], the *Cdkl5* +/− hearts showed higher levels of malondialdehyde (MDA), an end product of lipid peroxidation, consistent with a condition of oxidative stress (Figure 5B). Interestingly, UBQ supplementation restored MDA levels to those of the control condition in *Cdkl5* +/− mice (Figure 5B), an alteration that is indicative of decreased ROS production.

To investigate whether reduced ROS production elicited by UBQ supplementation improves cell oxidative stress, we analyzed the levels of the DNA damage marker γH2AX and of lamin B1 in cardiomyocyte nuclei. Interestingly, we found that nuclear γH2AX intensity was higher in the myocardium of *Cdkl5* +/− mice in comparison with wild-type (*Cdkl5* +/+) mice (Figure 5C,D), and UBQ supplementation restored γH2AX to control levels in *Cdkl5* +/− cardiomyocytes (Figure 5C,D), suggesting ROS-dependent increased DNA damage in the absence of CDKL5. Similarly, lamin B1 nuclear intensity was increased in *Cdkl5* +/− cardiomyocytes in comparison with wild types (Figure 5E,F). UBQ supplementation restored lamin B1 levels in *Cdkl5* +/− cells to wild-type levels (Figure 5E,F). The trend of lamin B1 expression in *Cdkl5* +/− cardiomyocytes was confirmed through Western blot analysis of heart homogenates (Figure 5G). As previously reported [19], we found that PARP1 levels and the LC3II/LC3I ratio, which are indicative of increased oxidative stress and autophagosome formation, respectively, were increased in the heart of *Cdkl5* +/− mice in comparison with wild types (Figure S4C,D). Confirming the beneficial antioxidant CoQ10 effect, UBQ treatment reduced PARP1 levels and, even if not significantly, the LC3-II/LC3-I ratio in *Cdkl5* +/− mice (Figure S4C,D)

## 3. Discussion

### 3.1. Increased ROS Cellular Levels in CDKL5-Deficient Cells

There is an increasing amount of experimental evidence that oxidative stress is involved in the neuropathology of several neurodevelopmental disorders, as well as cardiovascular diseases [53,54]. Here, we found that CDKL5 deficiency is associated with increased ROS levels in a neuronal cell type such as the SH-SY5Y human neuroblastoma. ROS are by-products of normal cell activity by many cellular compartments and play an important role in signaling pathways. Increased levels of ROS can occur from increased ROS production but also from decreased repair or removal processes.

We found that excessive production of ROS depleted GSH levels and increased DNA damage, as measured by the GSH/GSSG ratio and γH2AX intensity, respectively. Therefore, it is logical to hypothesize that an imbalance between ROS production and elimination by the antioxidant systems, in favor of ROS formation, occurs in SH*-CDKL5-*KO cells. Moreover, the finding that there are no differences in cell oxygen consumption between wild-type and SH*-CDKL5-*KO cells suggests that increased ROS production does not lead to damaged mitochondrial respiratory capacity in CDKL5-deficient cells. Since mitochondria are not the only source of cellular ROS, we can hypothesize that other cellular compartments/enzymes, such as NADPH oxidases (NOXs), may be responsible for the increased ROS accumulation in the absence of CDKL5. Since mitochondria have a high scavenging capacity for radicals [55], it is possible to assume that the increased ROS levels observed in CDKL5-deficient cells are efficiently counteracted at the mitochondrial level, to the extent that they have no effect on the functionality of the respiratory chain. Due to the complexity of the biochemical networks necessary to maintain the redox balance in cells, appropriate cellular studies will be needed to better understand the key events/pathways which trigger ROS accumulation in CDKL5-deficient cells.

By addressing the relationship between increased ROS production and cell senescence/survival in CDKL5-deficient cells, we have shown for the first time that CDKL5-deficient cells have an increased cellular level of endogenous lamin B1, and that high lamin B1 levels correlate with nuclear shape alterations. Our finding that treatment with the antioxidant CoQ10 restores both lamin B1 levels and nuclear shape alterations confirms the correlation of these alterations with increased ROS production. In support of the involvement of increased ROS production in the CDKL5 KO nuclear phenotype, we found that the antioxidant luteolin exerted a similar beneficial effect on nuclear shape in CDKL5-deficient cells. Our finding is in agreement with the recently proposed role of lamin B1 as a general marker and mediator of ROS-induced cell senescence [46,56]. In mammalian cells, structural changes in the nucleus are primarily governed by the nuclear lamina, an intermediate filament meshwork composed of A- and B-type lamins. Along with the perinuclear cytoskeleton and chromatin [57,58], the nuclear lamina regulates nuclear properties, including stiffness, size and shape. Published observations have shown that defects in lamin B1 lead to a change in nuclear morphology [46,56,59,60]. Consistent with our finding, increased lamin B1 levels and altered nuclear morphology have been described in the brain of the R6/1 mouse model of Huntington’s Disease [56]. However, decreased levels of lamin B1 also result in nuclear morphology alterations in Alzheimer’s [61] and Parkinson’s [62] neurons, suggesting that proper lamin B1 levels are necessary to maintain a correct neuronal nuclear morphology and that they correlate with cell survival.

Lamin-dependent nuclear remodeling is an important mechanism that also contributes to heart aging and dysfunction [63]. Interestingly, we observed increased lamin B1 levels in the *Cdkl5* +/− heart, which is characterized by typical signs of heart aging, including increased ROS production [18]. Therefore, increased lamin B1 levels might be considered as a new marker of oxidative stress in Cdkl5-deficient cells.

### 3.2. Treatment with CoQ10 Prevents ROS Cellular Accumulation and DNA Damage in CDKL5-Deficient Cells

CoQ10 has been found to be clinically effective against oxidative stress in many neurodevelopmental disorders, including Rett syndrome [40]. Many reports have examined the beneficial effects of CoQ10 in aging and age-related disorders [64,65] as an antioxidant, since, in its reduced form, CoQ10 acts directly as a membrane radical scavenger. In the current study, we found that CoQ10 not only prevents ROS cellular accumulation but significantly decreases, or even prevents, DNA damage and consequent cell senescence/death in SH*-CDKL5-*KO cells. It is worth noting that in vivo UBQ supplementation restores DNA damage in cardiac myocytes from *Cdkl5* +/− mice. The protective effect of CoQ10 against oxidative DNA damage has already been reported in the literature [66,67,68] and is interpreted as being associated with antioxidant activity or modulation of DNA repair enzymes. On the other hand, modulation of the intracellular redox environment by CoQ10 might be effective in promoting gene expression, possibly by inducing the expression of oxidative damage repair enzymes. Further studies are required to understand the mechanisms of CoQ10’s beneficial effects in CDKL5-deficient cells, especially in the context of its multiple biological functions.

### 3.3. Coenzyme Q10 Supplementation Prevents Nuclear DNA Damage and Restores Lamin B1 Levels in the Cdkl5 +/− Heart

The in vivo absorption and efficacy of CoQ10 is highly dependent on its formulation [69]. Phytosome formulation of CoQ10, UBQ [42], exhibits ameliorated bioavailability in comparison to regular CoQ10 and has been reported to increase CoQ10 content in skeletal muscle [44]. We showed here that UBQ supplementation in drinking water restores cardiac lipid peroxidation in adult female *Cdkl5* +/− mice, suggesting a reduction in oxidative stress. The *Cdkl5* +/− hearts showed increased levels of the DNA damage marker γH2AX as well as of nuclear lamin B1. Interestingly, both DNA damage and nuclear lamin B1 levels were restored in the *Cdkl5* +/− heart by treatment with CoQ10, suggesting that the antioxidant effect of CoQ10 may reduce or even prevent some of the damage caused by free radicals.

Our results have shown that there is a beneficial effect of UBQ supplementation at the cardiac level. However, despite finding an increase in plasma CoQ9 after CoQ10 levels in wild-type UBQ-treated mice, we did not observe a similar significant increase in cardiac tissue, suggesting a tissue-specific regulation of CoQ in mice [70]. Increased endogenous CoQ9 after CoQ10 supplementation is consistent with evidence demonstrating that, in rodents, prolonged CoQ10 dietary supplementation increases CoQ9 in many tissues [71]. On the other hand, CoQ10 supplementation in UBQ-treated *Cdkl5* +/− mice did not result in a significant increase in plasma CoQ levels, suggesting a decreased absorption of this compound. Indeed, gastrointestinal disturbances are reported as a common clinical characteristic of CDKL5 deficiency [6]. The cardiovascular protective effect of CoQ10 supplementation is described extensively in the literature (for a review see [72,73]). It is generally accepted that elevated levels of oxidized low-density lipoproteins (LDLs) are a risk factor for cardiovascular disease (CVD), and it is known that reduced CoQ10 protects human LDL from lipid peroxidation [74]. Interestingly, Takahashi and colleagues have shown that a CoQ10 reductase on the outer surface of liver cells can help maintain the reduced state of extracellular CoQ10 and thus prevent LDL oxidation [75]. In this context, we can argue that the small increase in CoQ10 plasma level detected in treated mice may be responsible for the significant decreased oxidative stress status observed in the cardiac tissue of UBQ-treated *Cdkl5* +/− mice. In addition, the two-week supplementation period might not be sufficient to detect a markable CoQ10 accumulation at the cardiac level [76]; further to this, UBQ administration through drinking water may not ensure optimal intake. Future studies using higher doses of UBQ or longer treatment periods, and/or changing the method of supplementation, may be helpful to assess whether a tissue accumulation effect of CoQ10 exists, and whether there is a correlation between an increase in CoQ10 in heart tissue and the therapeutic benefit in CDD.

## 4. Materials and Methods

### 4.1. Cell Lines and Treatments

Human neuroblastoma cell line SH-SY5Y, deriving from The European Collection of Authenticated Cell Cultures (Sigma-Aldrich, St. Louis, MO, USA) and the *CDKL5* knockout (KO) SH-SY5Y neuroblastoma cell line (SH*-CDKL5-*KO; [18]), were maintained in Dulbecco modified Eagle medium (DMEM, Thermo Fisher Scientific, Waltham, MA, USA) supplemented with 10% heat-inactivated Fetal Bovine Serum (FBS, Thermo Fisher Scientific, Waltham, MA, USA), 2 mM glutamine (Thermo Fisher Scientific, Waltham, MA, USA), and antibiotics (penicillin, 100 U/mL; streptomycin, 100 µg/mL, Thermo Fisher Scientific, Waltham, MA, USA), in a humidified atmosphere of 5% CO_2_ at 37 °C. The cell medium was replaced every 3 days and the cells were sub-cultured once they reached 90% confluence. For the UBQ treatment, the cells were grown for 24 h in complete culture medium supplemented with 100 nM CoQ10 Phytosome (UBIQSOME, UBQ, Indena S.R.L, Milan, Italy) or with Phytosome alone (the sunflower lecithin matrix as vehicle), which were provided by Indena S.p.A., Milan, Italy. For the luteolin treatment, the cells were grown for 24 h in complete culture medium supplemented with 10 µM luteolin (LUT, Tocris, Ellisville, MO, USA) or with vehicle (0.1% DMSO). For the mitochondrial oxygen consumption analyses in differentiated cells, the cells were plated in a 10 cm culture dish at a density of 1 × 10^6^ cells in a culture medium supplemented with 10% FBS. Twenty-four hours after cell plating, cell differentiation was induced by adding 10 μM retinoic acid (RA; Sigma-Aldrich) each day for 5 days.

### 4.2. Measurement of ROS

Oxidative stress was measured in intact cells using the reactive oxygen species indicator 2′,7′-dichlorodihydrofluorescein diacetate (DCF-DA, Thermo Fisher Scientific, Waltham, MA, USA), as previously described [77]. Briefly, SH-SY5Y and SH*-CDKL5-*KO neuroblastoma cells were seeded in 96-well plates at 4 × 10^4^ cells/well. After a 24 h period to allow adhesion, the cells were incubated with 100nM UBQ or Phytosome vehicle dissolved in complete medium for 24 h at 37 °C in 5% CO_2_. After this time, the cells were incubated with 10 μM DCF-DA in complete medium for 30 min. Subsequently, the cells were washed with Hank’s balanced salt solution (HBSS), and the fluorescence value in each well was measured (λexc = 485 nm; λem = 535 nm) with a plate reader (Enspire, Perkin Elmer, Shelton, CT, USA). The fluorescence emission was normalized in terms of protein content using the Lowry method.

### 4.3. Mitochondrial Oxygen Consumption Assay

The cellular oxygen consumption in intact cells was measured through polarography, using an oxygraph chamber (Instech Mod. 203, Plymouth Meeting, PA, USA), as reported in [78]. Briefly, SH-SY5Y and SH*-CDKL5-*KO neuroblastoma cells were washed in NaCl 0.9% and trypsinized; cells were collected and centrifuged, and the pellet was resuspended in complete medium. The cell suspension was put in the oxygraph chamber, and the oxygen consumption rate was measured in the basal condition and in presence of oligomycin A, carbonyl cyanide 4-(trifluoromethoxy) phenylhydrazone (FCCP), and Rotenone plus AntimycinA. The result was then normalized to total protein content.

### 4.4. Immunocytochemistry

For the immunocytochemistry analyses, the cells were plated onto poly-D-lysine-coated slides in a six-well plate at a density of 2.5 × 10^5^ cells per well in culture medium supplemented with 10% FBS. The day after, the cells were treated with 100 nM UBQ or vehicle for 24 h, fixed in a 4% paraformaldehyde solution at 37 °C for 30 min and processed for lamin B1 immunocytochemistry. For the γH2AX analyses, the cells were fixed in absolute methanol at −20 °C for 7 min and processed for immunocytochemistry. For the immunofluorescence studies on cell lines and cardiac tissue, the following antibodies were used: primary antibodies—recombinant rabbit monoclonal anti-lamin B1 (1:1000; Invitrogen—Thermo Fisher Scientific, Waltham, MA, USA) and rabbit polyclonal anti-γH2AX (phospho Ser139) (1:1000, Abcam, Cambridge, UK); secondary antibody—AlexaFluor 555-conjugated anti-rabbit (1:200, Thermo Fisher Scientific, Waltham, MA, USA). The nuclei were counterstained with DAPI (40,6-diamidino-2-phenylindole)-Fluoromount-G (SouthernBiotech, Birmingham, AL, USA).

### 4.5. Apoptotic and Mitotic Index

For the determination of the apoptotic and mitotic index, the nuclei were stained with DAPI (40,6-diamidino-2-phenylindole)-Fluoromount-G (SouthernBiotech, Birmingham, AL, USA). Fluorescence images were taken with an Eclipse TE 2000-S microscope equipped with a DS-Qi2 digital SLR camera (Nikon Instruments Inc., Melville, NY, USA). Apoptotic cell death was assessed by manually counting the number of pyknotic nuclei and apoptotic bodies and expressed as pyknotic index, i.e., number of apoptotic cells over the total cell number. The number of mitotic cells was assessed by manually counting the cells in prophase (chromosomes condensed and visible), metaphase (chromosomes lined up at the metaphase plate), and anaphase/telophase (chromosomes pulled toward the opposite poles), and expressed as mitotic index, i.e., number of mitotic cells over the total cell number.

### 4.6. Circularity Index Evaluation

Starting from 20× magnification images of SH-SY5Y and SH*-CDKL5-*KO neuroblastoma cells treated with 100 nM UBQ or vehicle for 24 h, the area of the DAPI-stained interphase nuclei was manually drawn using the Image Pro Plus software (version 6, Media Cybernetics, Silver Spring, MD, USA) measurement function and expressed in μm^2^. The nuclear circularity index was calculated as reported in [79] with the following equation: circularity = 4A/πM^2^, where A is the area and M is the length of the major axis of each nucleus. Circularity has a maximum value of 1 and diminishes as the nuclear shape becomes increasingly convoluted. Approximately 200 nuclei were analyzed from each sample.

### 4.7. Nuclear Deformation Analysis

SH-SY5Y and SH*-CDKL5-*KO neuroblastoma cells treated with 100 nM UBQ or vehicle for 24 h were immunostained for lamin B1, counterstained with DAPI, and fluorescence images were taken with an Eclipse TE 2000-S microscope equipped with a DS-Qi2 digital SLR camera (Nikon Instruments Inc., Melville, NY, USA). Based on previous studies [80], the nuclear morphological classification was divided into three types: invagination, when nuclei showed one clear lamin B1 invagination; evagination, when nuclei exhibited one clear lamin B1 protrusion from the nuclear lamina; and aberration, when nuclei presented a combination of more than one invagination, evagination, or additional nuclear abnormalities. Regular nuclei were considered those without any of these deformations. The relative number of nuclear deformations was quantified and expressed as a percentage of the total number of lamin B1 positive nuclei. A total of 200 nuclei were analyzed from each sample.

### 4.8. Glutathione Assay

The glutathione (GSH) levels in SH-SY5Y and SH*-CDKL5-*KO cells were quantified using a bioluminescent GSH/GSSG-Glo^®^ kit (Promega, Madison, WI, USA), following the manufacturer’s instruction. Briefly, neuroblastoma cells were seeded in 96-well plates at 4 × 10^4^ cells/well. After 24 h to allow adhesion, the cells were incubated with 100 nM UBQ or vehicle dissolved in complete medium for 24 h at 37 °C in 5% CO_2_. After this time, the cells were lysed with 25 μL GSH or GSSG lysis reagents, and luciferin generation reagent was added (50 μL), followed by luciferin detection regent (100 μL). GSH and GSSG standards were used to generate calibration curves. The luminescent signal was measured with a Glomax microplate reader (Promega, Madison, WI, USA), and the GSH/GSSG ratio was calculated as [(net total glutathione RLU − net GSSG RLU)/(net GSSG RLU)] × 2, where RLU stands for Relative Light Units.

### 4.9. Superoxide Determination

The mitochondrial ROS production was evaluated in SH-SY5Y and SH*-CDKL5-*KO cells using the MitoSOX^TM^ Green (Thermo Fisher Scientific, Waltham, MA, USA) fluorescent probe as previously described [81], with minor modifications. Briefly, 10 × 10^3^ cells were seeded in a µ-Slide 8 Well (Ibidi, Martinsried, Germany) and incubated overnight to allow adhesion. Then, the cells were washed with HBSS and treated with 5 µM MitoSOX^TM^Green (λ excitation = 488 nm; λ emission = 510 nm) dissolved in complete DMEM for 30 min. After this time, the cells were washed twice with HBSS, and the images were acquired using a Nikon 81 Clsi confocal microscope (Nikon Instruments Inc., Melville, NY, USA). Fluorescence intensity was obtained using the ImageJ v1.54k software tool.

### 4.10. Animal Husbandry

The mice used in this work derive from the *Cdkl5* −/Y strain in the C57BL/6N background developed in [12] and backcrossed in C57BL/6J for three generations. Heterozygous *Cdkl5* +/− females were produced and genotyped as previously described [12]; age-matched wild-type *Cdkl5* +/+ littermate controls were used for all experiments. The day of birth was designated as postnatal day (P) zero, and animals with 24 h of age were considered as 1-day-old animals (P1). After weaning (P21-23), mice were housed three to five per cage on a 12-h light/dark cycle in a temperature- and humidity-controlled environment with food and water provided ad libitum. The animals’ health and comfort were controlled by the veterinary service. Experiments were carried out on a total of 30 adult (6–8-month-old) *Cdkl5* KO mice (*Cdkl5* +/+ n = 12; *Cdkl5* +/− n = 18). The study protocols complied with EU Directive 2010/63/EU and with Italian law (DL 26, 4 March 2014) and were approved by the Italian Ministry of Health (protocol n 375/2024-PR). All efforts were made to minimize animal suffering and to keep the number of animals used to a minimum.

### 4.11. In Vivo Treatments

For in vivo treatment, 6–8-month-old *Cdkl5* +/+ and *Cdkl5* +/− female mice were provided with drinking water containing UBQ or vehicle (Phytosome) for two weeks. The daily intake of UBQ for each mouse was equivalent to ~500 mg of UBQ (corresponding to 100 mg of CoQ10) per kilogram of body weight. Considering an average daily consumption of 5 mL of drinking water and an average body weight of 30 g per mouse, drinking water was prepared with a concentration of 3 mg/mL of UBQ. The solutions were renewed three times a week. At the end of the treatment, the mice were weighed and put under deep anesthesia through inhalation of 2% isoflurane in pure oxygen. Blood samples were collected, and the mice were sacrificed through cervical dislocation.

### 4.12. Heart Dissection

The hearts were quickly removed, cleaned from the surrounding structures, and thoroughly washed in PBS to remove all blood, then weighed. The hearts were quickly frozen in isopentane, cooled in liquid nitrogen, and stored at –80 °C until used for immunohistochemistry and Western blot analyses.

### 4.13. γH2AX and Lamin B1 Immunohistochemistry

For immunohistochemistry procedures, the frozen hearts were cut with a cryostat (Histo-Line Laboratories, Pantigliate, MI, Italy) into 7 µm thick sections mounted on super frost slides. Sections were post-fixed via immersion in ice-cold methanol/ethanol (1:1) and permeabilized with 0.2% TritonX-100 in PBS. Furthermore, 2% BSA in PBS was used as a blocking reagent. Sections were incubated overnight with recombinant rabbit monoclonal anti-lamin B1 antibody (1:500; Invitrogen—Thermo Fisher Scientific, Waltham, MA, USA) and rabbit polyclonal anti-γH2AX (phospho Ser139) antibody (1:300, Abcam, Cambridge, UK), washed with PBS, and subsequently incubated for 2 h at room temperature with goat anti-rabbit IgG (H+L) (1:200, Alexa Fluor™488, Invitrogen—Thermo Fisher Scientific, Waltham, MA, USA) or goat anti-rabbit IgG (H+L) (1:200, Alexa Fluor™ 555, Invitrogen—Thermo Fisher Scientific, Waltham, MA, USA) secondary antibodies. The sections were mounted with DAPI (40,6-diamidino-2-phenylindole)-Fluoromount-G (SouthernBiotech, Birmingham, AL, USA).

### 4.14. Cdkl5 mRNA Detection

The In Situ Hybridization (ISH) for the *Cdkl5* RNA was performed on 7 µm thick heart sections with the Base Scope^®^ technology (Biotechne, Minneapolis, MN, USA) following the manufacturer’s protocol, using a 1ZZ probe designed on the *Cdkl5* exon 4 [82]. Detection was performed using the TSA Cyanine 3 Plus Fluorescein Evaluation Kit (Perkin Elmer, Waltham, MA, USA). 

### 4.15. Image Acquisition and Measurements

Fluorescence images were taken with an Eclipse TE 2000-S microscope equipped with a DS-Qi2 digital SLR camera (Nikon Instruments Inc., Melville, NY, USA). Starting from 40× magnification images, the perimeter of the nucleus was traced using the DAPI counterstaining as a guide to define the nuclear area of each cell, and the intensity of γH2AX and lamin B1 staining within each area was then quantified by determining the sum intensity of all positive (bright) pixels within the area. Approximately 100 nuclei were analyzed from each sample. For the quantification of *Cdkl5* mRNA, starting from 20× magnification images, the number of positive fluorescent signals was manually counted using the point tool of the Image Pro Plus software (Media Cybernetics, Silver Spring, MD, USA) and expressed per µm^2^.

### 4.16. Quantification of CoQ9 and CoQ10 Levels in Plasma and Heart Homogenates

Total plasma CoQ9 and CoQ10 content was measured through high-performance liquid chromatography (HPLC) as reported by [83]. Firstly, 200 µL of plasma was diluted 1:1 with bi-distilled water and transferred into a tube containing seven volumes of a n-hexane/ethanol mixture (5:2). Then, the tubes were vortexed for 2 min and centrifuged at 1700 rcf for 10 min. The upper layer from each sample was collected, and a second extraction was performed. The n-hexane solutions were dried out in glass tubes by nitrogen flux, and the dry extracts were resuspended in ethanol-FeCl_2_ 0.05%. 20 µL of samples were injected into a two-pump HPLC system equipped with photodiode array detector (Agilent, Santa Cruz, CA, USA) and a C18 column (Kinetex, Phenomenex, 5 µm 100 A, 150 × 4.6 mm), using an ethanol/water mobile phase (97:3, *v*/*v*) at a 0.8 mL/min flow rate. CoQ9 and CoQ10 peaks were detected at 275 nm by comparison with standards. Quantitation was performed by interpolation of the area under the curve using a calibration curve. CoQ7 was used as an internal standard. The results were normalized to the plasma volume. Total CoQ9 and CoQ10 levels were also measured in heart tissue homogenates. Briefly, heart tissue was homogenized using a Turrax; the homogenate was then subjected to 15 strokes in a glass potter and filtered with gauze. The resulting homogenate was extracted for CoQ9 and CoQ10 as described above. All procedures were carried out at 4 °C. The CoQ content was normalized to the protein content.

### 4.17. Measurement of Lipid Peroxidation

Lipid peroxidation in the heart tissue homogenates from *Cdkl5* +/− and *Cdkl5* +/+ female mice was assessed by measuring the biomarker Malondialdehyde (MDA) as previously described [84]. Briefly, the quantification of MDA was performed through reaction with thiobarbituric acid (TBA), and the measurement of the TBA-MDA adduct was carried out at 535 nm using a Jasco V-750 spectrophotometer. 1,1,3,3-tetramethoxypropane (Sigma-Aldrich, St. Louis, MO, USA) was used as standard.

### 4.18. Western Blot Analysis

For the preparation of protein extracts, SH-SY5Y and SH*-CDKL5-*KO cells or ventricular tissue from 6–8-month-old *Cdkl5* +/+ and *Cdkl5* +/+ female mice were homogenized in ice-cold RIPA buffer (50 mM Tris–HCl, pH 7.4, 150 mM NaCl, 1% Triton-X100, 0.5% sodium deoxycholate, 0.1% SDS) supplemented with 1 mM PMSF and with a 1% protease and phosphatase inhibitor cocktail (Sigma-Aldrich, St. Louis, MO, USA). The protein concentration for both cell and tissue extracts was determined using the Bradford method [85]. Equivalent amounts (50 μg) of protein were subjected to electrophoresis on a 4–12% Mini-PROTEAN^®^ TGX™ Gel (Bio-Rad Laboratories, Inc., Hercules, CA, USA) and transferred to a Hybond ECL nitrocellulose membrane (Amersham—GE Healthcare Life Sciences, Chicago, IL, USA). The following primary antibodies were used: rabbit monoclonal anti-lamin B1 (1:1000; Invitrogen—Thermo Fisher Scientific, Waltham, MA, USA), rabbit polyclonal anti-γH2AX (phospho Ser139; 1:1000, Abcam, Cambridge, UK), mouse monoclonal anti-GPX4 (1:500; SantaCruz Biotechnology, Dallas, TX, USA), rabbit polyclonal anti-PARP1 (1:1000; Abcam, Cambridge, UK), rabbit polyclonal anti-LC3B (1:1000; Thermo Fisher Scientific, Waltham, MA, USA), mouse monoclonal anti-SOD1 (1:500; SantaCruz Biotechnology, Dallas, TX, USA), rabbit polyclonal anti-GAPDH (1:5000; Sigma-Aldrich, St. Louis, MO, USA), and mouse monoclonal anti-Vinculin (1:1000, Santa Cruz Biotechnology, Dallas, TX, USA). An HRP-conjugated goat anti-rabbit IgG (1:5000, Jackson ImmunoResearch Laboratories, Inc., West Grove, PA, USA) secondary antibody was used. The densitometric analysis of digitized Western blot images was performed using Chemidoc XRS Imaging Systems and the Image Lab^TM^ Software (Bio-Rad Laboratories, Inc., Hercules, CA, USA); this software automatically highlights any saturated pixels of the Western blot images in red. Images acquired with exposition times that generated protein signals out of a linear range were not considered for the quantification. Repeated measurements of the same samples were performed, running from two to four different gels. The signal of one sample (internal control) was used to perform a relative analysis of the antigen expression of each sample on the same gel. We considered the control signal as 100 and assigned a value to the other sample as a percentage of the control. Data analysis was performed by averaging the signals obtained in two to four gels for each individual sample.

### 4.19. Statistical Analysis

The statistical analysis was performed using GraphPad Prism (version 9, San Diego, CA, USA). The values are expressed as means ± standard error (SEM). The significance of results was obtained using the two-tailed Student’s *t*-test, one-way ANOVA, or two-way ANOVA followed by Tukey or Fisher’s LSD post hoc tests as specified in the figure legends. A probability level of *p* < 0.05 was considered to be statistically significant. The confidence level was taken as 95%.

## 5. Conclusions

A large body of evidence suggests that oxidative injury is important in either the primary or downstream secondary pathophysiological mechanisms underlying many neurologic disorders [86]. Therefore, a major therapeutic opportunity lies in preventing the production of oxidants that cause direct brain injury. Therapies aimed at boosting antioxidant defenses or reducing pro-oxidant production using free radical scavengers or antioxidants may be efficacious in preventing, ameliorating, or arresting many neurologic diseases. However, the biochemistry of oxidative pathobiology is complex, and optimum antioxidant therapeutic options may vary and need to be tailored to individual diseases.

These results demonstrate that CoQ10 could be an important antioxidant for ameliorating oxidative stress-mediated dysfunction in CDKL5-deficient cells. We believe that future studies aimed at understanding the benefits of CoQ10-mediated blockade of oxidative stress in the Cdkl5-deficient brain will provide the rationale for the use of CoQ10 as supplemental therapy in CDD.

## Figures and Tables

**Table 1:** CoQ9 and CoQ10 plasma levels in *Cdkl5* +/+ and *Cdkl5* +/− mice treated with UBQ 500 mg/kg/day for 14 days compared to vehicle-treated *Cdkl5* +/+ and *Cdkl5* +/− mice. * *p* < 0.05, ** *p* < 0.01, n.s. = not significant (two-tailed Student’s *t*-test).

Q9 (pmol/mL Plasma)			
	*Cdkl5* +/+	*Cdkl5* +/−	*p*
vehicle	178.9 ± 13.3 (n = 6)	192.9 ± 20.3 (n = 9)	n.s.
UBQ	471.3 ± 136.6 (n = 7)	232.7 ± 27.1 (n = 10)	*
*p*	**	n.s.	
Q10 (pmol/mL plasma)			
	*Cdkl5* +/+	*Cdkl5* +/−	*p*
vehicle	68.9 ± 16.9 (n = 5)	37.9 ± 2.3 (n = 5)	n.s.
UBQ	126.0 ± 9.2 (n = 5)	42.6 ± 9. 8 (n = 8)	*
*p*	*	n.s.	

**Table 2:** CoQ9 and CoQ10 cardiac levels in *Cdkl5* +/+ and *Cdkl5* +/− mice treated with UBQ 500 mg/kg/day for 14 days compared to vehicle-treated *Cdkl5* +/+ and *Cdkl5* +/− mice. n.s. = not significant (two-tailed Student’s *t*-test).

Q9 (pmol/mg Protein)			
	*Cdkl5* +/+	*Cdkl5* +/−	*p*
vehicle	644.1 ± 74.6 (n = 6)	712.2 ± 64.4 (n = 10)	n.s.
UBQ	574.9 ± 75.5 (n = 7)	754.5 ± 54.4 (n = 10)	n.s.
*p*	n.s.	n.s.	
Q10 (pmol/mg protein)			
	*Cdkl5* +/+	*Cdkl5* +/−	*p*
vehicle	83.3 ± 9.5 (n = 6)	95.5 ± 9 (n = 10)	n.s.
UBQ	75.1 ± 11.2 (n = 7)	100.6 ± 10.2 (n = 10)	n.s.
*p*	n.s.	n.s.	

## Data Availability

The datasets analyzed during the current study are available from the corresponding author upon reasonable request.

## Supplementary Materials

The following supporting information can be downloaded at: https://www.mdpi.com/article/10.3390/ijms26052204/s1.
