# Supplementary material for: Beneficial Antioxidant Effects of Coenzyme Q10 in In Vitro and In Vivo Models of CDKL5 Deficiency Disorder
Source: Int J Mol Sci. 2025 Feb 28;26(5):2204. doi: 10.3390/ijms26052204 (PMC11900000; doi:10.3390/ijms26052204)
Supplement: Supplementary file 1 [file ijms-26-02204-s001.zip › ijms-3504324-supplementary/Supplementary Figures_rev .pdf]

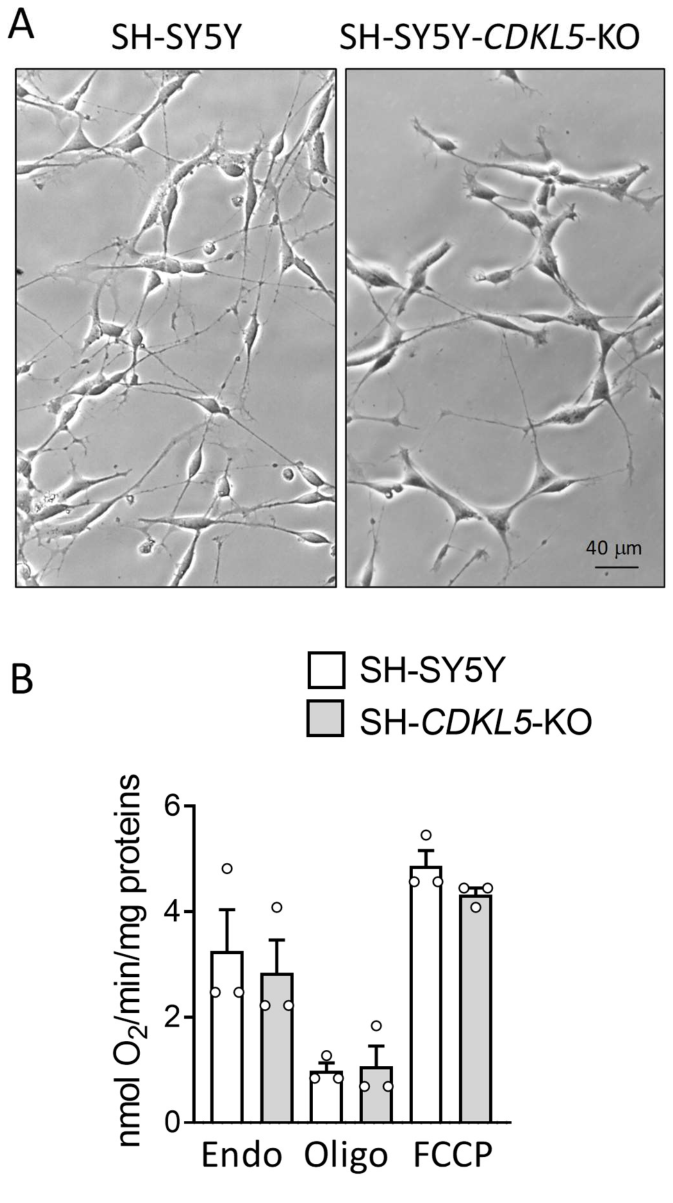

Supplementary Figure 1.

Mitochondrial Oxygen consumption rate in differentiated SH-SY5Y and SH-*CDKL5*-KO neuroblastoma cells. (**A**) Representative phase-contrast images showing the morphology of SH-SY5Y parental cells and SH-*CDKL5*-KO after 5 days of treatment with retinoic acid (RA; 10 μM). (**B**) Histogram shows oxygen consumption rate in intact differentiated SH-SY5Y and SH-*CDKL5*-KO neuroblastoma cells (endogenous respiration "Endo"), in the presence of 1 μM oligomycin A (Oligo) and 0.25–1 μM carbonyl cyanide 4-(trifluoromethoxy) phenylhydrazone (FCCP). Data are expressed as nanomoles of oxygen per minute, normalized to protein content. Values represent means ± SEM of at least 3 independent experiments. Tukey test after one-way ANOVA.

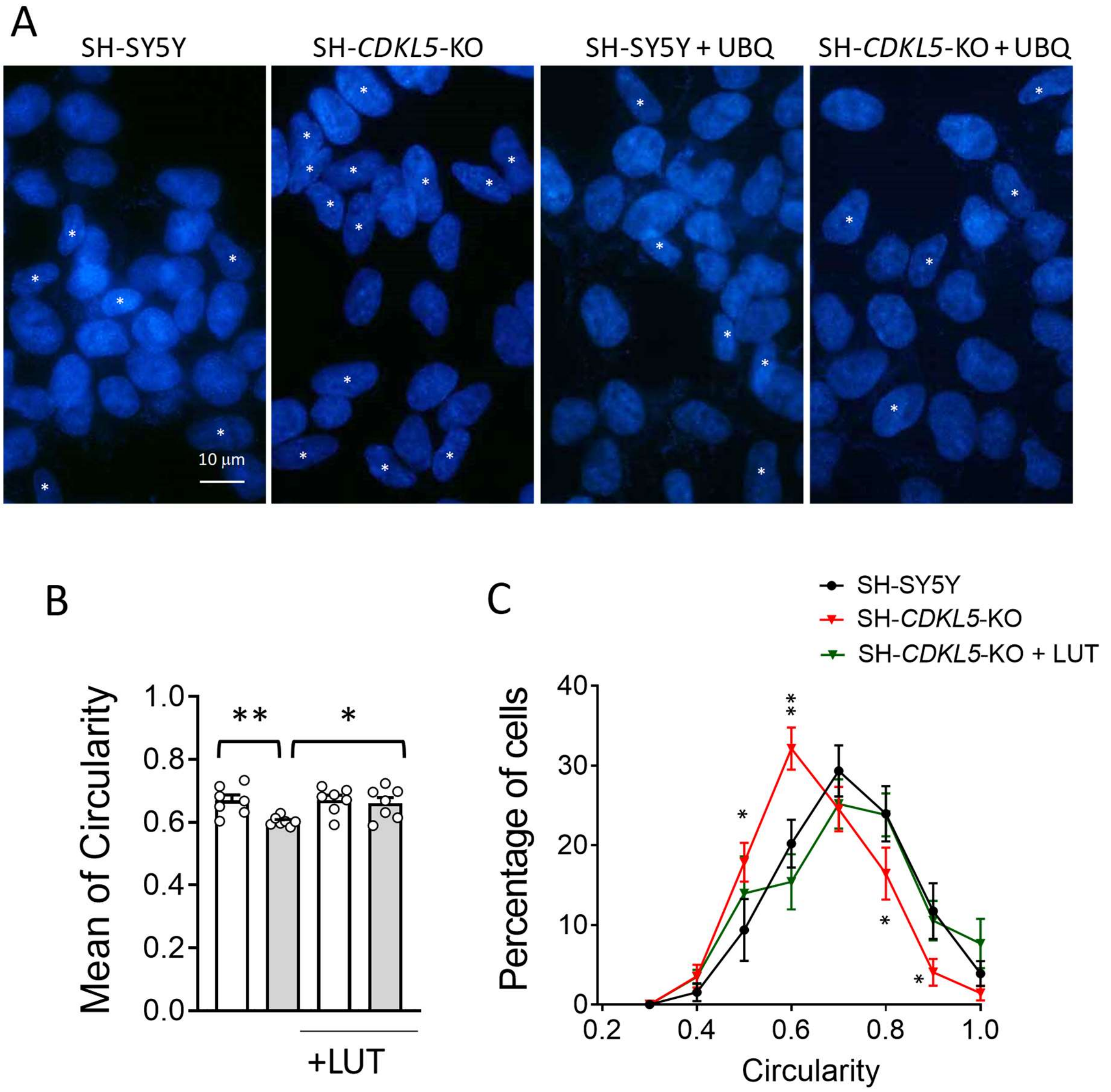

Supplementary Figure 2.
Effect of treatment with UBQ or Luteolin on nuclear shape in SH-*CDKL5*-KO cells**.** (**A**) Representative fluorescence images of nuclear shape of DAPI-stained nuclei in SH-SY5Y and SH-*CDKL5*-KO cells treated with 100 nM CoQ10 Phytosome (UBQ) or vehicle for 24 h. Asterisks indicate nuclei with the circularity index ≤ 0.6. (**B,C**) Morphometric analysis of nuclei from SH-SY5Y and SH-*CDKL5*-KO cells treated with Luteolin (LUT, 1 μM) or vehicle for 24 h. The histogram in (**B**) shows quantification of the mean circularity index. Distribution analysis of nuclear circularity in (**C**). Approximately 140 nuclei were analyzed for each experimental group. Data are shown as percentage of nuclei displaying a specific circularity index for each experimental group. The results in (**B,C**) are presented as means ± SEM. * $p < 0.5$; ** $p < 0.01$. Fisher's LSD test after two-way ANOVA.

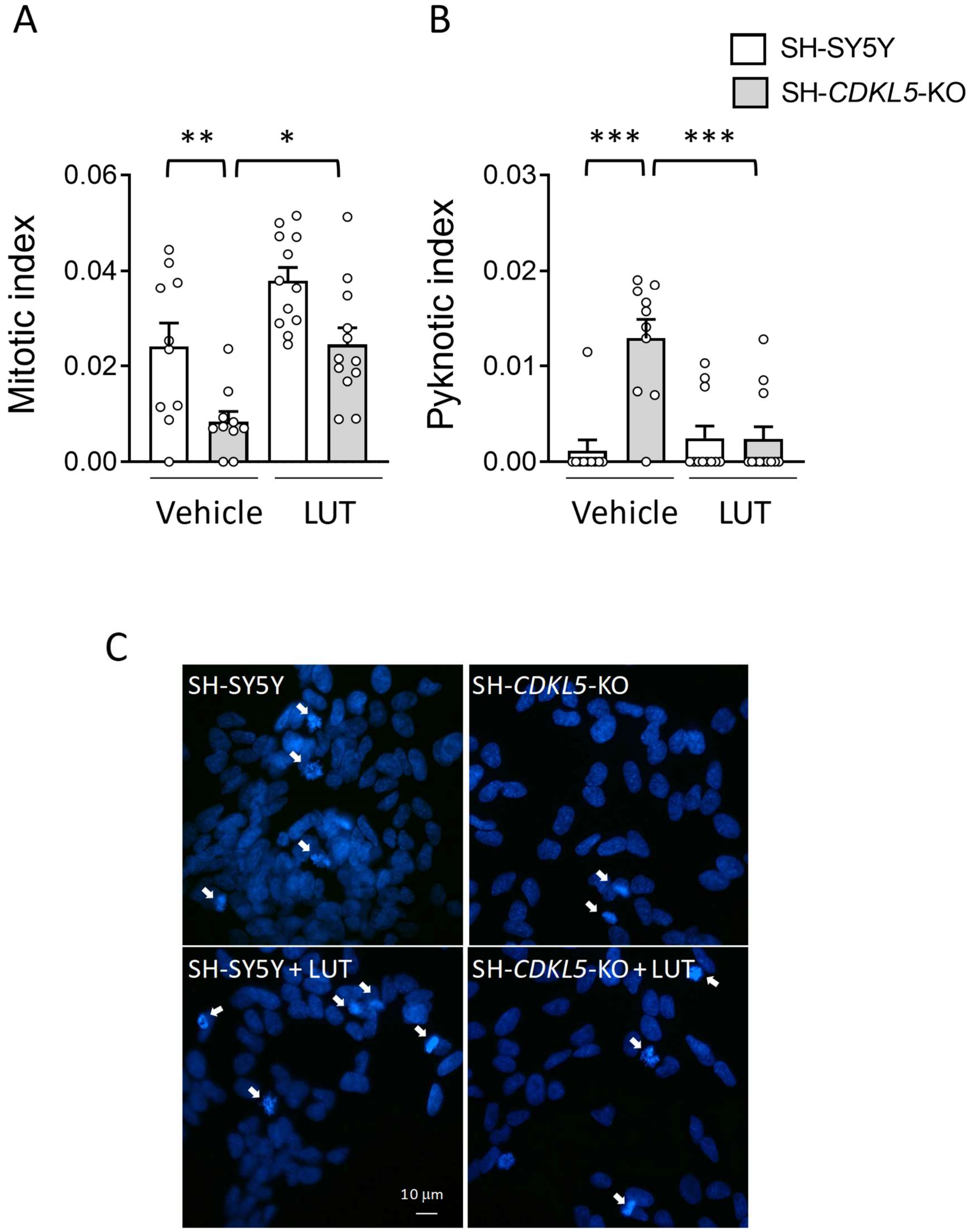

Supplementary Figure 3.

Effect of treatment with Luteolin on neuronal proliferation and survival in SH-SY5Y and SH-*CDKL5*-KO cells. (**A,B**) Evaluation of the number of mitotic (**A**) and apoptotic (**B**) cells in vehicle-treated SH-SY5Y and SH-*CDKL5*-KO cells, and in SH-SY5Y and SH-*CDKL5*-KO cells treated with Luteolin (LUT; 1 μM) for 24 h. Data are expressed as mitotic or pyknotic index, i.e., number of mitotic or apoptotic cells over total cell number. Approximately 120 cells were analyzed for each experimental group. (**C**) Representative images showing mitotic nuclei (white arrows) in each experimental condition. Values represent means ± SEM of 3 independent experiments. * $p < 0.05$; ** $p < 0.01$; *** $p < 0.001$ (Tukey test after two-way ANOVA).

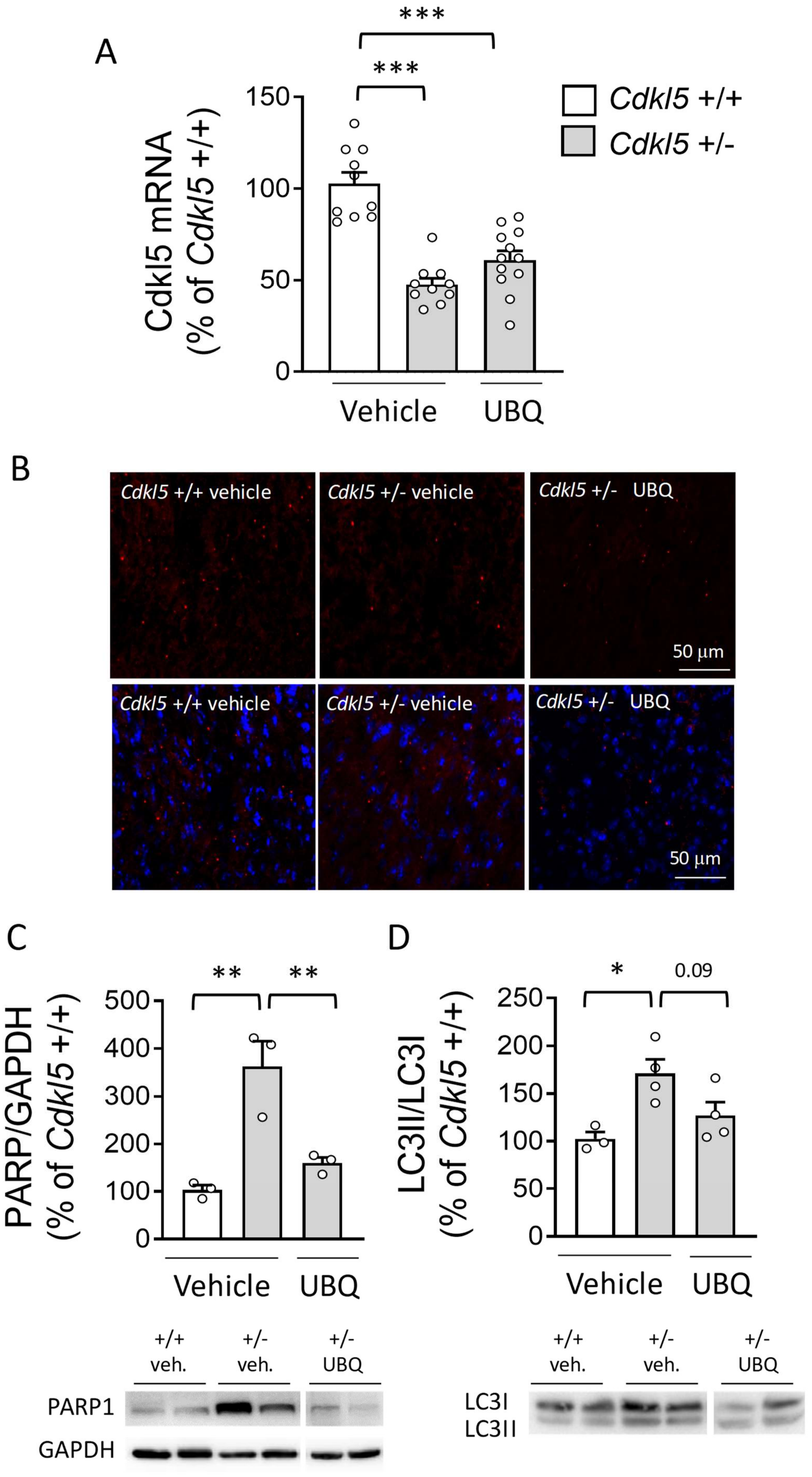

A
***
***
150
100
50
0
Cdkl5 mRNA
(% of Cdkl5 +/+)
Cdkl5 +/+
Cdkl5 +/-
Vehicle
UBQ
B
Cdkl5 +/+ vehicle
Cdkl5 +/- vehicle
Cdkl5 +/- UBQ
50 µm
Cdkl5 +/+ vehicle
Cdkl5 +/- vehicle
Cdkl5 +/- UBQ
50 µm
C
**
**
500
400
300
200
100
0
PARP/GAPDH
(% of Cdkl5 +/+)
Vehicle
UBQ
+/+ veh.
+/- veh.
+/- UBQ
PARP1
GAPDH
D
*
0.09
250
200
150
100
50
0
LC3II/LC3I
(% of Cdkl5 +/+)
Vehicle
UBQ
+/+ veh.
+/- veh.
+/- UBQ
LC3I
LC3II

Supplementary Figure 4.

(**A,B**) Fluorescence in situ hybridization (ISH) for *Cdkl5* mRNA in mouse heart sections. Quantification of the levels of *Cdkl5* mRNA expression (**A**) in the heart of six-month-old *Cdkl5* +/+ and *Cdkl5* +/- female mice treated with vehicle and *Cdkl5* +/- mice treated with UBQ 500 mg/Kg/day for 14 days. Values are presented as means ± SE. ***P < 0.001 (Fisher's LSD test after two-way ANOVA). Representative images of ISH for *CDKL5* mRNA in ventricular tissue sections of one mouse for each experimental group. *Cdkl5* mRNA (red), DAPI (blue; in the lower panels) (**B**). (**C,D**) Western blot analysis of poly(ADP-ribose) polymerase 1 (PARP1) and autophagy marker light chain 3 (LC3) levels in extracts of heart tissue from *Cdkl5* +/+ and *Cdkl5* +/− mice treated as in A. The histogram in (**C**) shows PARP1 protein levels normalized to GAPDH. Examples of immunoblots for PARP1 and GAPDH of two animals from each experimental group are presented in the lower panel. The histogram in (**D**) shows the ratio of LC3II/LC3I. Examples of immunoblots for LC3 of two animals from each experimental group. Data in (**A,C,D**) are expressed as percentages of vehicle-treated *Cdkl5* +/+ mice; values are presented as means ± SEM; * $p < 0.05$; ** $p < 0.01$; *** $p < 0.001$ (Tukey test after one-way ANOVA).
