# Supplementary material for: Beneficial Antioxidant Effects of Coenzyme Q10 in In Vitro and In Vivo Models of CDKL5 Deficiency Disorder
Source: Int J Mol Sci. 2025 Feb 28;26(5):2204. doi: 10.3390/ijms26052204 (PMC11900000; doi:10.3390/ijms26052204)
Supplement: Supplementary file 1 [file ijms-26-02204-s001.zip › ijms-3504324-supplementary/Supplementary Tables_rev.pdf]

**Q10** *(pmol/mg protein)*	***SH-SY5Y***	***SH-CDKL5-KO***	***p***
vehicle	108.6 ± 3.94	116.8 ± 10.6	n.s.
UBQ	232.2 ± 19.7	193.3 ± 40.7	n.s.
p	**	(*)	

Supplementary Table 1. CoQ10 levels in SH-SY5Y and SH-*CDKL5*-KO cells treated with 100 nM CoQ10 Phytosome (UBQ) for 24 h compared to vehicle-treated SH-SY5Y and SH-CDKL5-KO cells. (*) $p = 0.061$; **$p < 0.01$, n.s. = not significant (Fisher's test after two-way ANOVA).

	SH-SY5Y	*SH-CDKL5-KO*	*SH-CDKL5-KO UBQ*
SOD1	100 ± 0.83	111 ± 4.27 (n.s.)	118 ± 6.28 (n.s.)
PARP1	100 ± 14.6	110 ± 9.68 (n.s.)	113 ± 4.34 (n.s)

Supplementary Table 2. Quantification of superoxide dismutase 1 (SOD1) and Poly(ADP-ribose) polymerase 1 (PARP1) levels in protein extracts from SH-SY5Y and SH-*CDKL5*-KO cells and SH-*CDKL5*-KO cells treated with 100 nM CoQ10 Phytosome (UBQ) for 24 h. SOD1 and PARP1 protein levels were normalized to vinculin protein levels. n.s. = not significant (Tukey test after one-way ANOVA).
